# Equine Multinodular Pulmonary Fibrosis in association with asinine herpesvirus type 5 and equine herpesvirus type 5: a case report

**DOI:** 10.1186/1751-0147-54-57

**Published:** 2012-09-25

**Authors:** Helena Back, Anna Kendall, Rodrigo Grandón, Karin Ullman, Louise Treiberg-Berndtsson, Karl Ståhl, John Pringle

**Affiliations:** 1 Department of Virology, Immunology and Parasitology, The National Veterinary Institute, Uppsala, Sweden; 2 Institute of Veterinary, Animal and Biomedical Sciences, Massey University, Palmerston North, New Zealand; 3 Department of Biomedical Sciences and Veterinary Public Health. Division of Pathology, Pharmacology and Toxicology, Swedish University of Agricultural Sciences, Uppsala, Sweden; 4 Department of Clinical Sciences, Swedish University of Agricultural Sciences, Uppsala, Sweden

**Keywords:** Horse, Equine Multinodular Pulmonary Fibrosis, AHV-5, EHV-5, Lung

## Abstract

A standardbred gelding with a history of 10 days pyrexia and lethargy was referred to the Equine Hospital at the Swedish University of Agricultural Sciences in Uppsala, Sweden.

The horse had tachypnea with increased respiratory effort and was in thin body condition. Laboratory findings included leukocytosis, hyperfibrinogenemia and hypoxemia. Thoracic radiographs showed signs of pneumonia with a multifocal nodular pattern, which in combination with lung biopsy findings indicated Equine Multinodular Pulmonary Fibrosis (EMPF). EMPF is a recently described disease in adult horses with clinical signs of fever, weight loss and respiratory problems. The pathological findings include loss of functional pulmonary parenchyma due to extensive nodular interstitial fibrosis which has been related to infection with the equine herpesvirus type 5 (EHV-5). In this case, lung biopsy and tracheal wash samples tested positive for both asinine herpesvirus type 5 (AHV-5) and EHV-5 using PCR assays. The horse failed to respond to treatment and was euthanized for humane reasons. Postmortem examination confirmed the diagnosis of EMPF. This case suggests that not only EHV-5 alone should be considered in association with the development of this disease.

## Background

Equine herpesvirus type 5 (EHV-5) is one of the nine herpesviruses known to infect Equidae [1]. The family Herpesviridae is divided into three subfamilies: *Alphaherpesvirinae, Betaherpesvirinae* and *Gammaherpesvirinae*, where EHV-5 together with EHV-2 and EHV-7 (also called asinine herpes virus −2 [1]) are classified as equine gammaherpesviruses [2]. EHV-5 was first isolated in the seventies from two horses in quarantine in Australia with clinical signs of upper respiratory tract disease [3]. EHV-5 is recognized as a widespread finding in both nasal secretions and peripheral blood mononuclear cells of asymptomatic horses [4-7]. However, little is known about the potential role of EHV-5 causing clinically or subclinical infections associated with disease or poor performance in horses. One study has shown a higher prevalence of EHV-5 (as well as EHV-2) in horses with airway inflammation when compared to clinical healthy horses [8]. The asinine herpesviruses (AHV) 4 and 5 are also classified as gammaherpesviruses and are closely related to the equine gammaherpesviruses [9]. In horses AHV infection has only been described sporadically. Nonetheless, in a case–control study of horses with respiratory disorders or “poor performance syndrome” Fortier et al. [10] detected AHV-5 only in the clinical case group, which suggested that AHV-5 may play a clinically important role in horses.

There is also increasing evidence that EHV-5 may be associated with the occurrence of Equine Multinodular Pulmonary Fibrosis (EMPF), which was first described in 2007 as a disease in adult horses characterized by chronic, multifocal, fibrotic lung disease [11]. The clinical presentation of EMPF includes pyrexia, weight loss and respiratory signs ranging from mild tachypnea to marked dyspnea. Thoracic radiographs reveal an interstitial-to-nodular pulmonary pattern [12] and the most consistent hematological changes are neutrophilia and hyperfibrinogenemia [13]. Histological examination of lung tissue include multiple, well-demarcated nodular regions of pulmonary interstitial fibrosis with mixed inflammatory cell infiltration. Although the role of EHV-5 in EMPF is not fully determined, there is a strong circumstantial association of this virus with the pulmonary pathology presented in EMPF [11]. Similarly it appears that the gammaherpesvirus Murine herpesvirus 68 (MHV68) may be a co-factor for the development of a comparable presentation of pulmonary fibrosis seen in mice [14].

EMPF and infection with EHV-5 has been reported in both the USA [11,12,15] and Europe [16-18]. Some of the horses diagnosed with EMPF appeared to be infected with both EHV-5 and EHV-2 but co-infection with EHV-2 has not been further investigated [11,16,17]. By comparison, AHV-4 and AHV-5 in association with EMPF have only been described by Kleiboeker et al. where a group of donkeys with respiratory disease at necropsy demonstrated changes associated with interstitial pneumonia [9]. The present case report describes a standardbred gelding with clinical signs and lung pathology consistent with EMPF. What is striking in this case is the apparent co-infection with AHV-5 and EHV- 5. Both viruses were detected by PCR in samples from lung tissue and tracheal wash. While detection of AHV-5 in this horse with EMPF does not imply causation, its role as a pathogen in horses warrants further study, in particular as a co-factor with EHV-5 in the development of EMPF.

### Case presentation

A 4-year-old standardbred gelding weighing approximately 560 kg, was referred to the Equine Hospital at the Swedish University of Agricultural Sciences in Uppsala. Prior to presentation there was a 10-day history of fever (38.5-40°C), unwillingness to move, intermittent tachypnea and mild cough. No nasal discharge had been noted. The horse had been treated with procaine penicillin intramuscularly and acetylcysteine orally with no improvement. The gelding had been with the current owner at the same farm for three years prior to presentation. There was no history of contact with donkeys. The owner has given consent for publication of this clinical case.

### Physical examination and laboratory findings

On admission the horse was lethargic and tachypneic with shallow breathing. Clinical examination revealed a rectal temperature of 38.2°C, a heart rate of 48 beats per minute and a respiratory rate of 52 breaths per minute. The horse was in thin body condition and increased breathing sounds were present bilaterally over the thorax. Further diagnostic evaluation included venous and arterial blood sampling, endoscopy, chest radiography, ultrasound and finally percutaneous lung biopsy. On endoscopy there were multiple petechial hemorrhages generally distributed on the laryngeal mucosa and a mild accumulation of mucus in the trachea. A tracheal aspirate obtained via the endoscope was negative for bacterial growth under aerobic and anaerobic conditions. No cytology was performed on the tracheal aspirate.

Hematological and biochemical findings included: leukocytosis (17.7 x 10^9^/L reference range 5.5-12), with a mature neutrophilia (10.8 x 10^9^/L reference range 2–6.6), hyperfibrinogenemia (7.4 g/L reference range 1.8-4.2) and low serum creatinine (52 μmol/L reference range 88–145).

These findings suggested a systemic inflammatory process. The hypocreatinemia was likely related to the thin body condition and low muscle mass of the horse.

The arterial blood sample showed hypoxemia (Pao_2_ 51.75 mmHg, A-a gradient 42).

Thoracic radiographs showed a generalized mixed interstitial and nodular pattern in the lung parenchyma, and ultrasonographic examination revealed a diffuse roughening of the pleural surface, without signs of free fluid in the thorax.

A transthoracic lung biopsy was performed, under ultrasound-guidance, using a BARD Magnum^a^ on the right side of thorax following sedation with butorphanol (Butador®)^b^ and detomidine i.v. (Cepesedan®)^c^ and local anesthesia of the skin and deeper tissues at the biopsy site. Histological examination of the lung biopsy performed with hematoxylin and eosin (HE) staining demonstrated fibrosis with a widening of the interstitial space and presence of mature, intermittently hyalinated collagen. In the same area a mild to moderate infiltrate of inflammatory cells (mainly lymphocytes, a few macrophages and neutrophilic granulocytes) was present. Fibrosis was seen around smaller bronchioles and cuboidal cells lined alveoli. The epithelium of the bronchioles was intact and showed mild to moderate exocytosis. In alveoli and in some bronchioles a moderate to marked infiltrate of inflammatory cells mostly composed of degenerated neutrophils was observed. A histological diagnosis of chronic active interstitial fibrotic pneumonia was made.

No fungi were detected with periodic acid-shiff (PAS) staining and there was no bacterial growth obtained from culture of the biopsy under aerobic or anaerobic conditions.

According to a multi PCR analysis for respiratory viruses in horses^d^, a sample from nasal swab was negative for Equine Influenza Virus, EHV-1, EHV-4, Equine Rhinitis B Virus and Equine Arteritis Virus.

### Treatment and outcome

Initially the horse was treated with salbutamol (Ventoline®)^e^ 0. 7 mg/kg administrated via Aeromask^f^, and provided intranasal oxygen (15 L/min), dexamethasone (Dexadreson®)^g^ 0. 1 mg/kg bwt i.v. q 24 h and penicillin (Geepenil®)^h^ 27 167 IU/kg bwt i.v. q 8 h.

The horse did not respond to treatment and 3 days after admission started showing signs of severe dyspnea. Due to the clinical deterioration and the poor prognosis, the horse was humanely euthanized.

### Postmortem examination

**Figure 1:**
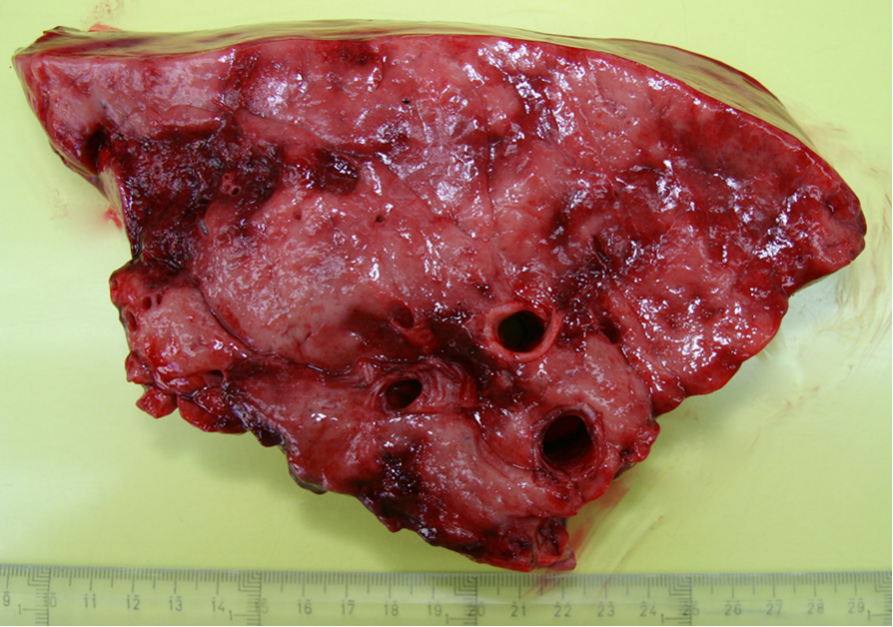
**The photo shows lung from the affected gelding. Fibrotic nodules are large, whitish, and multifocal to coalescing.** There are sharp borders between nodules and adjacent normal lung tissue.

At necropsy, multifocal to coalescing pale, firm, well-demarcated nodules varying in size from 2–10 cm were detected affecting all lung lobes (Figure 1) and only very small areas of normal parenchyma were visible. Further, the tracheobronchial lymph nodes were markedly enlarged; reaching approximately 8 x 3 cm.

**Figure 2:**
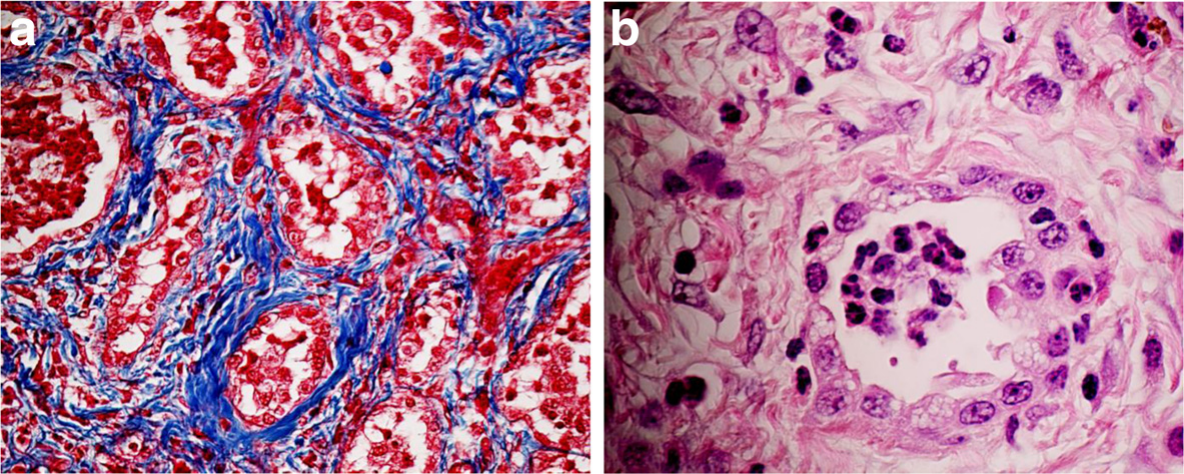
**a) Lung of the horse showing interstitial fibrosis (blue) around the alveoli.** Masson trichrome staining. **b**) Alveolus lined by cuboidal epithelial cells (type II pneumocytes) and surrounded by a fibrous stroma. The alveolar space filled by numerous inflammatory cells, primarily neutrophils. Hematoxylin and eosin staining.

Histological examination (Figure 2) of the lung tissue with hematoxylin-eosin (HE) and Masson´s trichrome stained sections demonstrated a moderate to severe, multifocal to coalescing interstitial fibrosis with presence of mature collagen. In the interstitial area mild to moderate infiltrates of inflammatory cells composed of lymphocytes, macrophages and neutrophils were observed whereas the airspace of the affected alveoli was lined by cuboidal epithelial cells (type II pneumocytes). The epithelium of bronchioles showed hypertrophy. Marked infiltrates of inflammatory cells, mostly composed of degenerated neutrophils, were detected in alveolar spaces and in some bronchioles.

### PCR and sequence analysis

**Figure 3:**
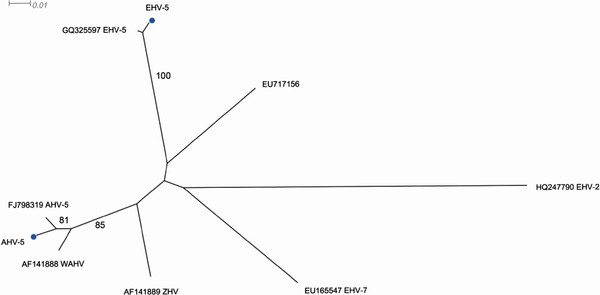
**Unrooted phylogram based on a 166 nucleotide fragment of the DNA polymerase gene from obtained AHV-5 and EHV-5 sequences together with selected related equine herpesvirus sequence from GenBank (virus/accession numbers: EHV-2/HQ247790, EHV-5/GQ325597, EHV-7/EU165547, AHV-5/FJ98319, Zebra Herpesvirus/AF141889, Wild Ass Herpesvirus (WAHV)/AF14188, and Eastern Kiang Herpesvirus/EU17156 incorrectly denominated EHV-9 in GenBank), constructed using the Neighbor-joining method and the Kimura-two-parameter model in SplitsTree4.** Numbers indicate the bootstrap values (1000 replicates) and only the values above 70% are shown in the figure. Branch distances are proportional to sequence distances. The figure indicates a strong relationship between the sequences obtained in this study (marked with blue circles) and preciously described representatives of EHV-5 and AHV-5, respectively.

The clinical signs, findings at necropsy and absence of other pathogens corresponded well with EMPF, and thus samples from lung tissue and tracheal wash were submitted for further virus examination. A broadly reacting nested consensus primer PCR, designed for detection of a wide variety of herpesviral genomes and targeting 215–315 base pair segment of the DNA polymerase was performed on DNA extract from samples of lung tissue and tracheal wash [19,20]. Viral nucleic acid was extracted using a magnetic bead separation method and subsequently amplified with the consensus primer PCR. Second round amplification products were separated in agarose gel with ethidium bromide and visualized under ultraviolet light. Bands with the approximate size of 200 base pairs were visible on the gel indicating the presence of herpesvirus. For sequence analysis the PCR products from the second round were reamplified and sequenced as previously described [19]. The 195 nt sequence obtained showed 98-100% nucleotide identity when compared with AHV-5 sequences available in GenBank using the blastn tool (http://www.ncbi.nlm.nih.gov/blast/Blast.cgi), and between 85–97% nucleotide identity with other available equine herpesviruses originating from domestic or wild equids. In order to gain more sequence information a primer pair designed for gammaherpesvirus also targeting the DNA polymerase gene and including the fragment produced by the consensus PCR was applied to the extracted DNA [21]. The PCR products were sequenced in both directions with the same primers. When comparing the sequence data, this new PCR product of 847 nt was found to be 99% identical to EHV-5 (GQ325597) whereas the part overlapping the consensus PCR fragment was only 87% identical to the previously obtained sequence. The results were confirmed by repeated PCR amplification and sequencing. Phylogenetic analysis was performed on the overlapping fragments of AHV-5 and EHV-5 and related equine herpesviruses sequences obtained from GenBank (virus/accession number: EHV-2/HQ247790, EHV-5/GQ325597, EHV-7/EU165547, AHV-5/FJ798319, Zebra Herpesvirus/AF141889, Wild Ass Herpesvirus (WAHV)/AF141888, and Eastern Kiang herpesvirus/EU717156 incorrectly denominated EHV-9 in GenBank) using Neighbour-joining and the Kimura-two-parameter model as implemented in SplitTree4 [22] clearly confirming the close relation between the sequences obtained with AHV-5 and EHV-5, respectively (Figure 3). 

## Conclusions

This case report describes a horse with clinical signs and histological changes within the lung that correspond with the recently described disease EMPF [11] that has been associated with infection by EHV-5. What was noteworthy in this clinical case was that AHV-5 was also found along with EHV-5, making it unclear whether one, or both in concert were responsible for the pulmonary disease.

AHV-5 and EHV-5 are two separate viruses. As can be shown in the phylogenetic tree (Figure 3) there is a close relationship between the AHV-5 sequence from the present case with reference sequences for AHV-5 and the WAHV. Albeit based on short fragment of the genome the phylogeny show a distinct difference between AHV-5 and EHV-5 even though they both belong to the gammaherpesvirus subfamily.

Pulmonary interstitial fibrosis is an uncommon disease in horses and has often been described as being idiopathic in origin [23,24]. In human medicine considerably more research has been conducted on the genesis of idiopathic pulmonary fibrosis (IPF) but the pathogenesis remains unclear. IPF may be related to epithelial cell injury, abnormal fibroproliferation, inflammation and deposition of extracellular matrix components. Several viruses, especially gammaherpesviruses, have been implicated as co-factors for initiating, promoting or exacerbating IPF [14,25-27]. While these studies do not prove total causality, the association between gammaherpesviruses and IPF like conditions in horses, mice and human is striking.

To the authors´ knowledge, this is the first report of EMPF in Sweden and also the first report with AHV-5 in horses in association with EMPF. Previous studies on EMPF appear to have focused their investigations on EHV-1, EHV- 2, EHV-4 and EHV-5 thus the co- presence of the AHV in some of those cases may also have occurred. However, even if this report indicates an association, further studies with inclusion of age and breed-matched control horses are needed to define the possible role of AHV-5 in the pathogenesis in EMPF and a baseline prevalence study could give valuable information about the distribution and importance of this pathogen. Furthermore, the development of a quantitative PCR for EHV-5 and AHV-5 would be of benefit to evaluate if one of the pathogens is present in larger quantity at the site of the lesions, possibly indicating an active role in the pathogenesis.

In conclusion, it appears that not only EHV-5 but also other equine gammaherpesviruses should be considered when investigating the cause/s of EMPF.

## Competing interests

There are no competing interests.

## Authors’ contributions

HB coordinated the work, contributed to the phylogenetic analysis, drafted the manuscript and contributed to final manuscript preparation. AK examined, sampled and treated the horse and contributed to drafting of the manuscript. RG performed the pathology and histology and contributed to drafting of the manuscript. KU carried out the molecular diagnostics and sequencing, contributed to the phylogenetic analysis and drafting of the manuscript. LTB contributed to the molecular diagnostics, and to drafting of the manuscript. KS contributed to the phylogenetic analysis, drafting of the manuscript and final manuscript preparation. JP contributed to drafting the manuscript and final manuscript preparation. All authors read and approved the final manuscript.
